# Supplementary material for: Emotional and Subsequent Behavioral Responses After Unethical Pro-Organizational Behavior: A Meta-Analysis Based Systematic Review
Source: Behav Sci (Basel). 2025 Sep 16;15(9):1266. doi: 10.3390/bs15091266 (PMC12467725; doi:10.3390/bs15091266)

Figure S1. Funnel plot for pride

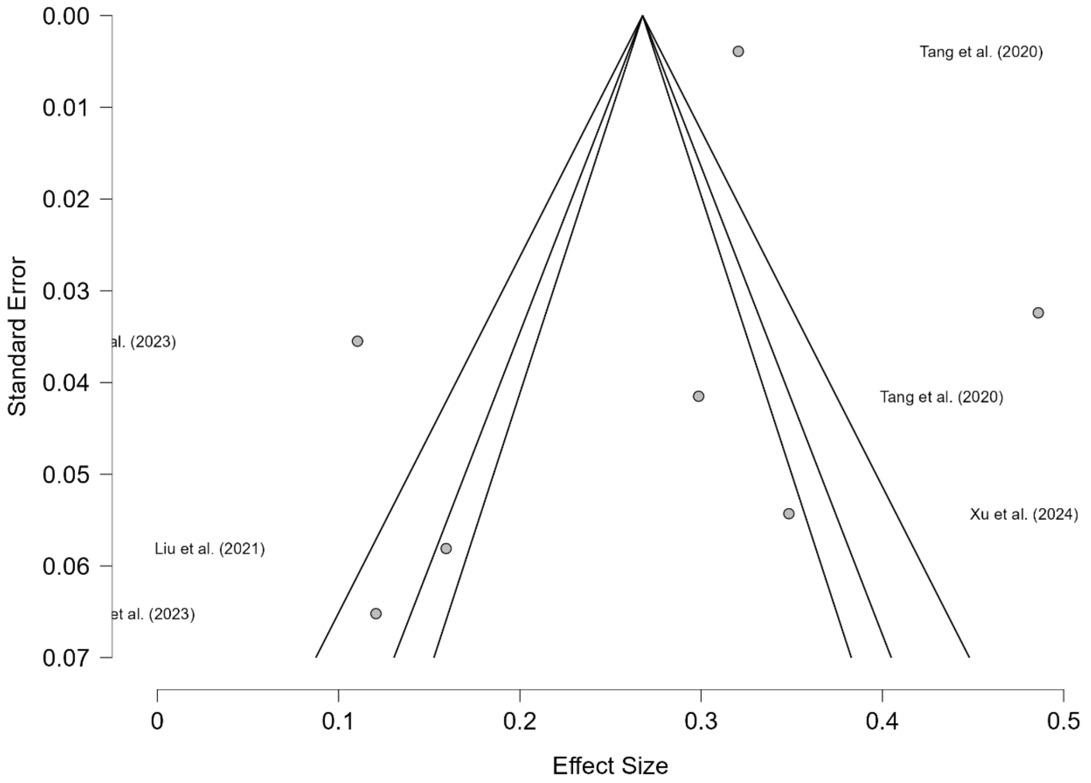

Figure S2. Funnel plot for guilt

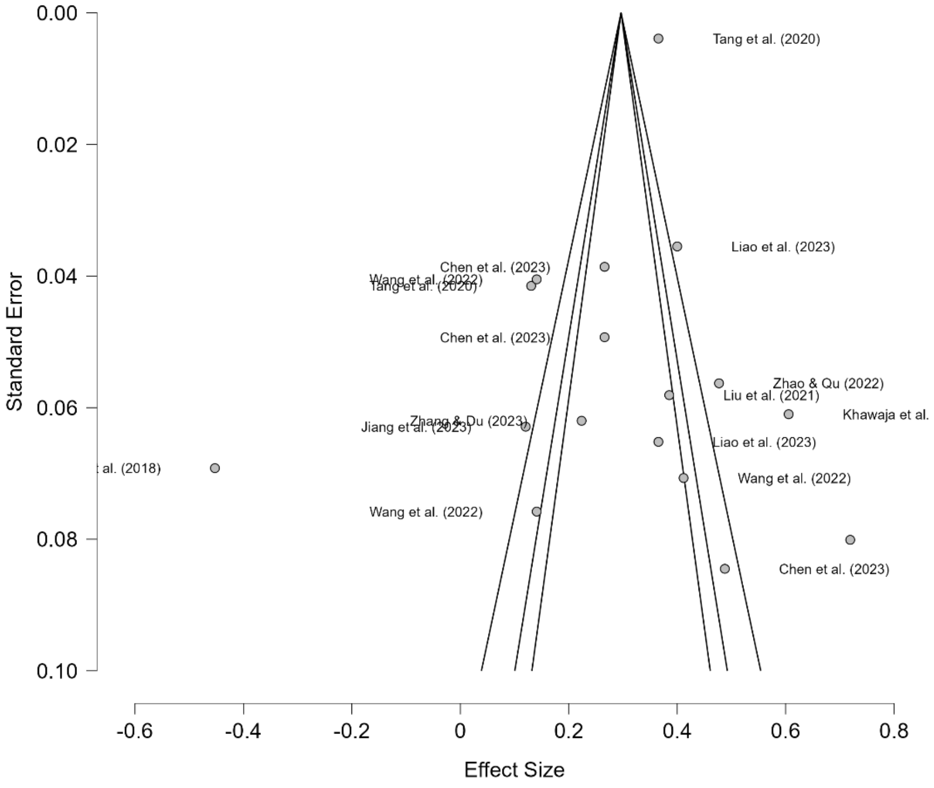

Figure S3. Funnel plot for shame

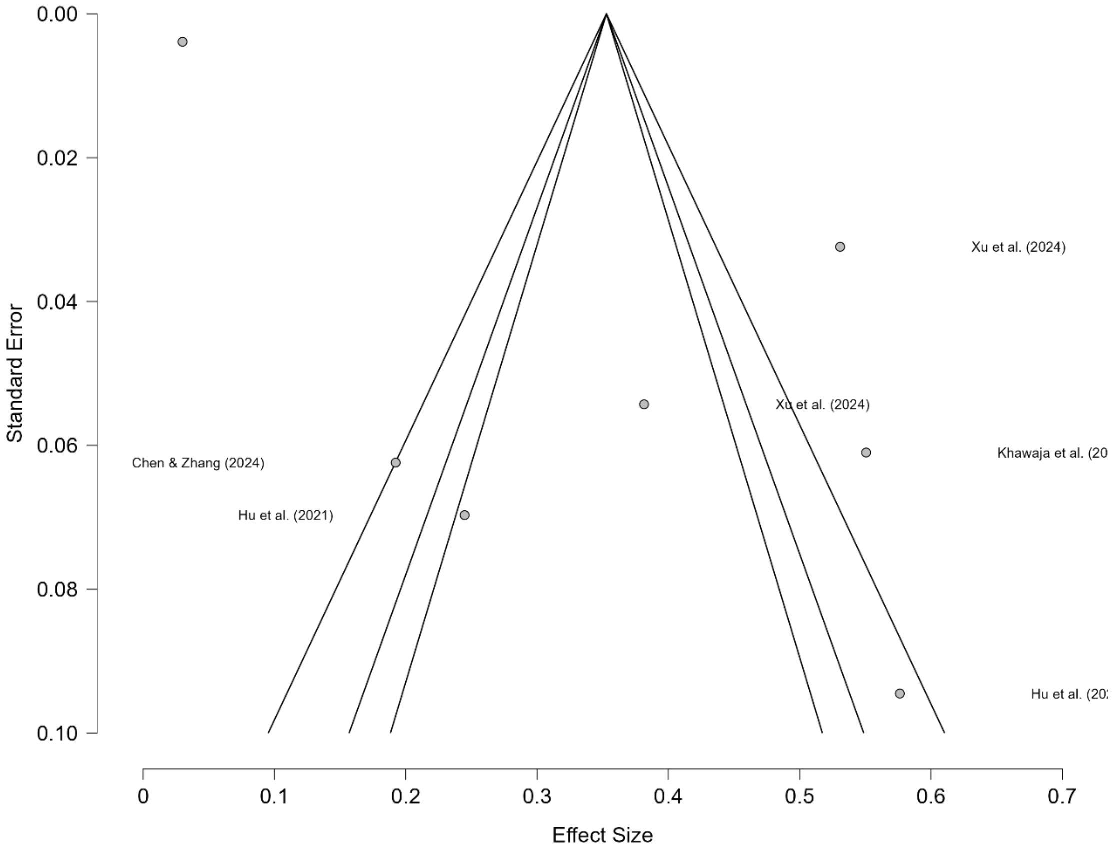

Figure S4. Funnel plot for anxiety

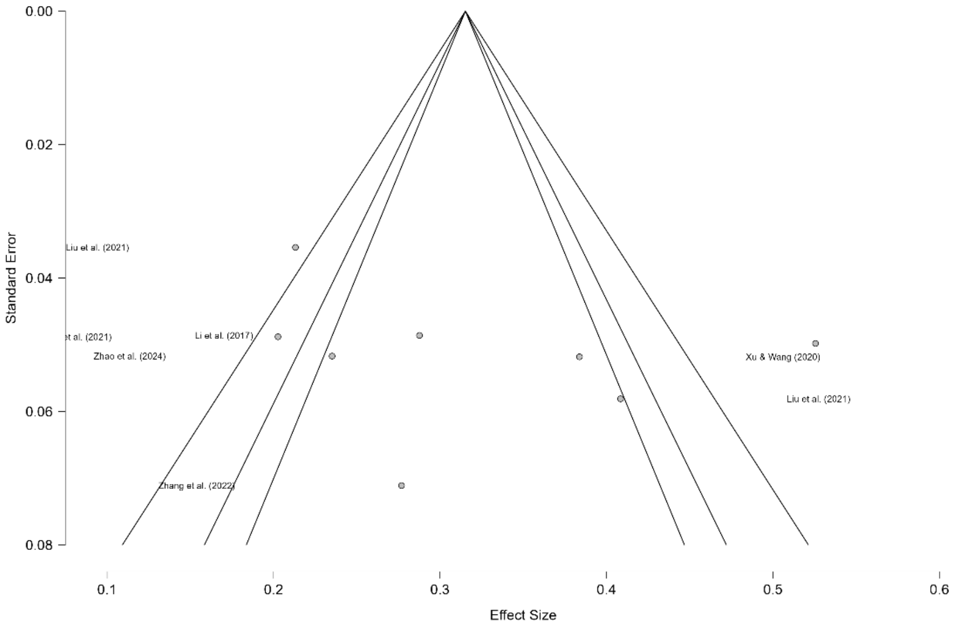

Figure S5. Funnel plot for emotional exhaustion

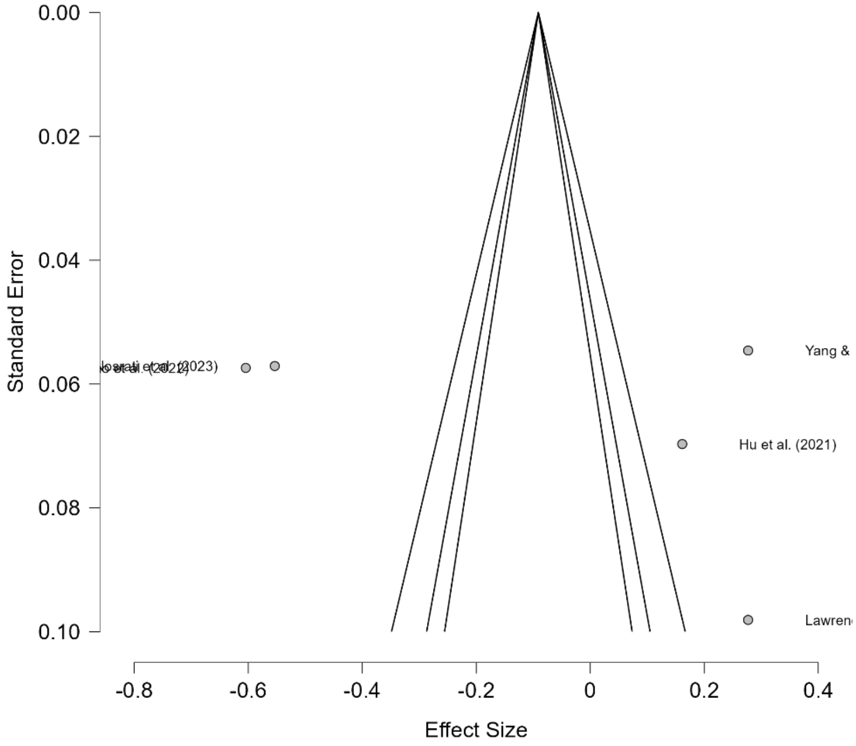

Figure S6. Funnel plot for psychological entitlement

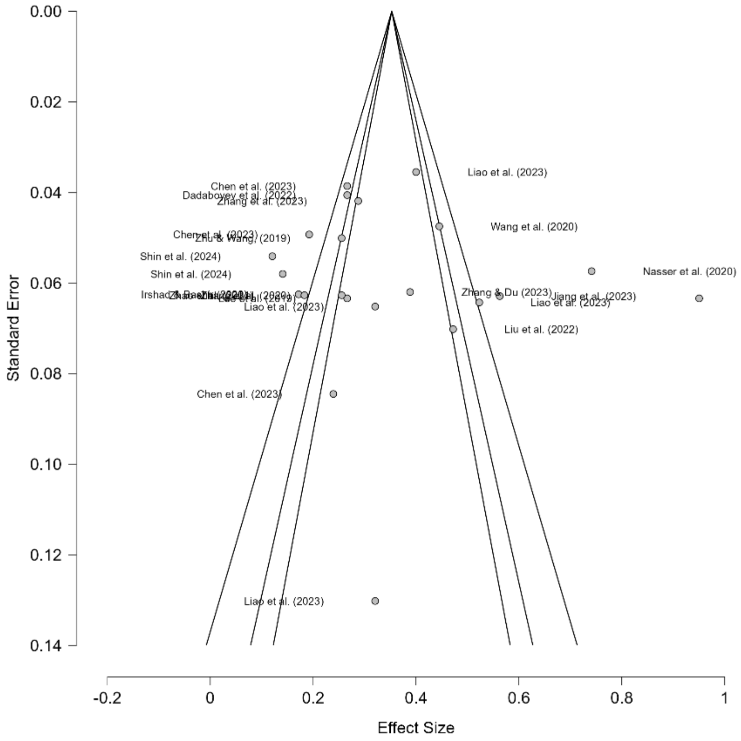

Figure S7. Funnel plot for moral licensing

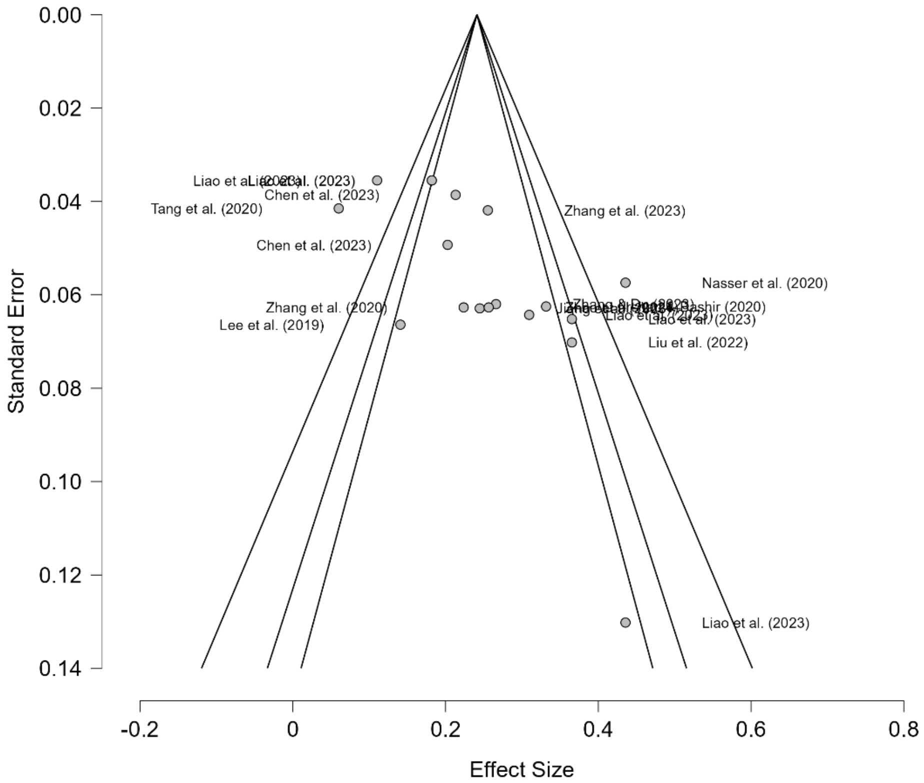

Figure S8. Funnel plot for moral slippery slope

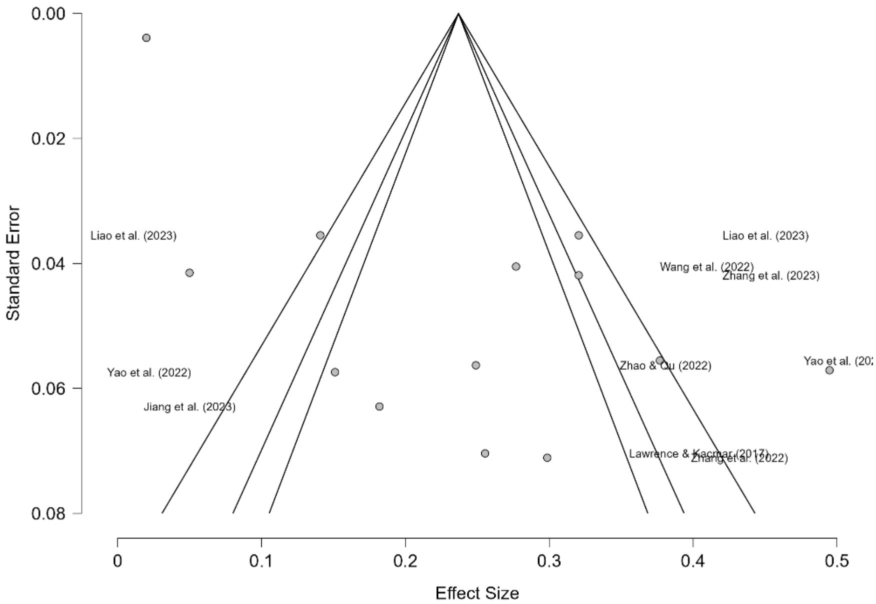

Figure S9. Funnel plot for moral cleansing

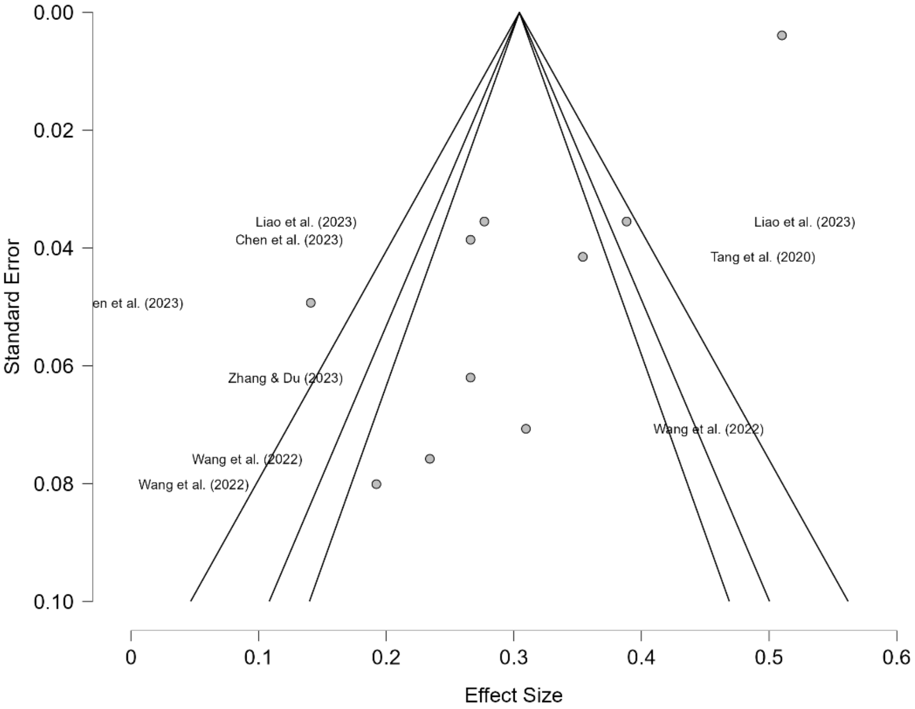

Figure S10. Funnel plot for Conscientiousness effect

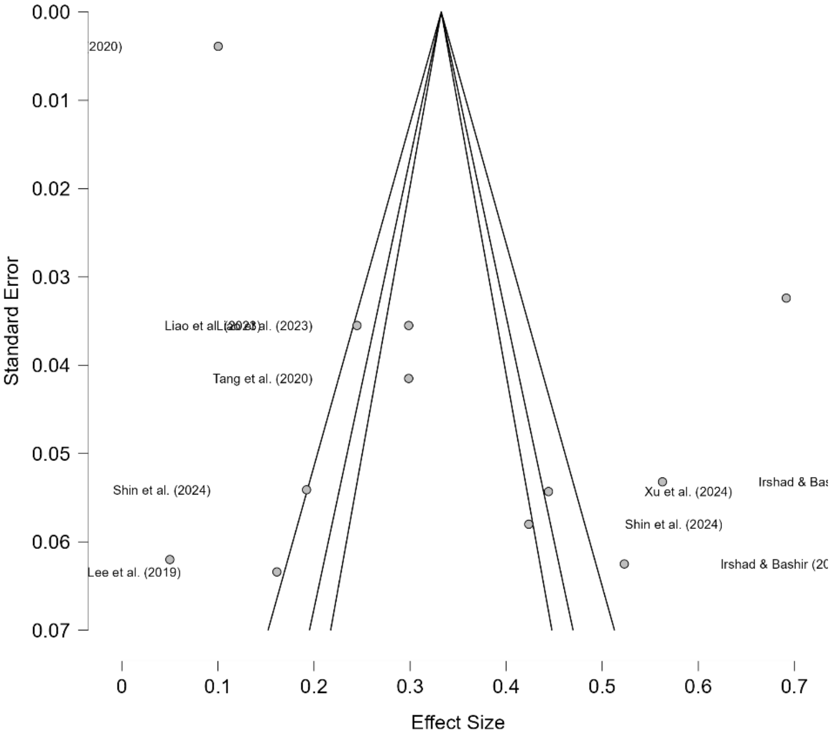

Figure S11. Funnel plot for Emotional rationalizing

effect

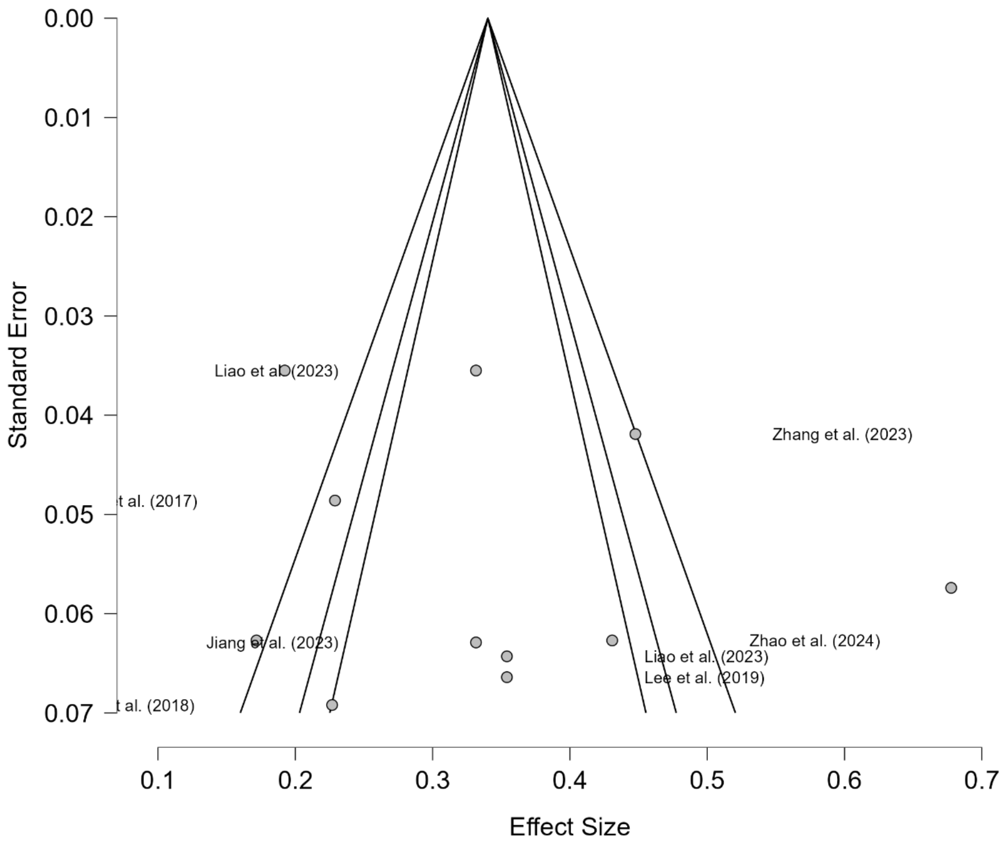

Supplement: Supplementary file 1 [file behavsci-15-01266-s001.zip › behavsci-3755737-supplementary.pdf]
